# Hydrogen-terminated diamond field-effect transistor with AlO_x_ dielectric layer formed by autoxidation

**DOI:** 10.1038/s41598-019-41082-8

**Published:** 2019-03-26

**Authors:** Yan-Feng Wang, Wei Wang, Xiaohui Chang, Xiaofan Zhang, Jiao Fu, Zhangcheng Liu, Dan Zhao, Guoqing Shao, Shuwei Fan, Renan Bu, Jingwen Zhang, Hong-Xing Wang

**Affiliations:** 1 Institute of Wide Band Gap Semiconductors, Xi’an Jiaotong University, Xi’an, &10049 PR China; 2 Shaanxi Key Lab of Information Photonic, Xi’an Jiaotong University, Xi’an, &10049 PR China; 3 Key Lab for Physical Electronics and Devices, Ministry of Education, School of Electronics and Information Engineering, Xi’an Jiaotong University, Xi’an, &10049 PR China

## Abstract

Fabrication of hydrogen-terminated diamond (H-diamond) field-effect transistor (FET) with AlO_x_ dielectric layer has been successfully carried out. The AlO_x_ layer was formed by auto-oxidizing 6 nm Al film in the air at room temperature, and a FET without AlO_x_ dielectric layer has also been fabricated for comparison. For both FETs, 100 nm Al layers were deposited as the gate electrodes, respectively. The leakage current density in FET with AlO_x_ dielectric layer was four magnitude orders lower than that without AlO_x_ dielectric layer at V_GS_ = −5 V, indicating that AlO_x_ dielectric layer could effectively reduce leakage current and prevent reverse I_D_ in I_D_ − V_DS_ caused by defects on diamond surface. Distinct pinch-off characteristic with p-type channel was observed in I_D_ − V_DS_ measurement. The threshold voltage was −0.4 V at V_DS_ = −15 V.

## Introduction

Diamond is an attractive material with many excellent properties such as good bio-compatibility, highest thermal conductivity (22 W/K·cm), large bandgap (5.45 eV), high theoretical breakdown voltage (>10 MV·cm^−1^), high carrier mobilities (electron: 4500 cm^2^ V^−1^ S^−1^, hole: 3800 cm^2^ V^−1^ S^−1^) etc., having potential applications in biology field, especially electronic devices such as metal oxide semiconductor field-effect transistors (MOSFETs) and metal semiconductor field-effect transistors (MESFETs)^1–8^, which can operate in high frequency, high power, high temperature. However, due to the high dopants activation energies (boron 380 meV and phosphorous 570 meV) in diamond at this stage, carrier densities are quite low at room temperature (RT), leading to the poor performance of MOSFET and MESFET based on diamond^8^. In order to overcome this issue, some groups try to use δ-doping technique in diamond. However, this technique was complex and the carrier mobility is not enough^9,10^. Fortunately, when diamond surface is terminated with C-H bonds by hydrogen plasma treatment, two-dimensional hole gases (2DHG) with 10^13^ cm^−2^ sheet carrier density can be accumulated on its surface^11^, by which H-diamond FET can be fabricated.

Up to now, many dielectric materials have been used in H-diamond FETs such as SiO_2_^12^, ZrO_2_^5^, Al_2_O_3_^13^, AlN^14^, TiO_x_^4^, HfO_2_^15^, LaAlO_3_^16^ and Ta_2_O_5_^17^. To fabricate gate dielectric layers in H-diamond MOSFETs, many methods have been used such as atomic layer deposition, metal organic chemical vapor deposition or magnetron sputtering techniques. However, these techniques are expensive, complex and may deteriorate 2DHG channel layer by high temperature or plasma etching, because 2DHG of H-diamond is thermally and chemically instable^18^. Therefore, researchers should simplify gate oxide deposition and protect the 2DHG channel layer during fabrication process.

In this work, H-diamond FET with AlO_x_ dielectric layer formed by auto-oxidizing in the air at RT was successfully fabricated. To authors’ knowledge, few investigations on H-diamond FETs using autoxidation AlO_x_ dielectric layer has been reported.

## Methods

**Figure 1:**
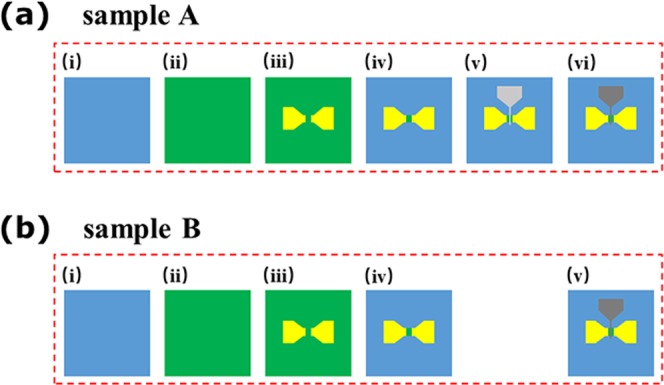
Fabrication process of the (**a**) sample A and (**b**) sample B, respectively.

Two 3×3 × 0.5 mm^3^ Ib-type single crystal diamonds (001) were used as substrates defined as sample A and B. Figure 1(a,b) showed the fabrication process of the H-diamond FET with AlO_x_ dielectric layer (sample A) and FET without AlO_x_ dielectric layer (sample B), respectively.

To remove non-diamond phase from diamond surfaces, sample A and B were cleaned by a mixed acid and then treated with a mixed alkali as our previous work^6^, shown as Fig. 1(a,b-i). After that, microwave plasma CVD system (AX5200 Seki Technotron Corp.) was used to grow 200 nm single crystal H-diamond on sample A and B, shown as Fig. 1(a,b-ii). The growth conditions has been shown in our previous report^7^. Then, source and drain Au electrodes were fabricated on sample A and B by photolithographic and electron beam evaporation (EB) techniques, shown as Fig. 1 (a,b-iii). The thickness and space of electrodes (L_SD_) were 100 nm and 20 μm, respectively. Thereafter, negative photoresist was used to coat on channels between source and drain electrodes by photolithographic technique. After that, UV/ozone was used on sample A and B to form isolation, shown as Fig. 1(a,b-iv). Then, gate patterns were formed on sample A and B by photolithographic technique. For sample A, 6 nm Al film was deposited on diamond surface with gate patterns by EB technique, and then sample A was oxidized in the air at RT for 48 hrs to form AlO_x_ dielectric layer, shown as Fig. 1(a-v). For comparison, sample B with gate patterns was placed in the air at RT for 48 hrs without 6 nm Al deposition on diamond surface. After 48 hrs done, 100 nm Al gate electrodes were deposited on sample A and B. The gate width (W_G_) and the gate length (L_G_) were 100 μm and 8 μm.

Micro-Raman, X-ray diffraction (XRD) and atomic force microscope (AFM) were used to characterize the samples, and the electrical properties of FETs with and without AlO_x_ were measured using RT probe system all in the air at RT.

## Results and Discussion

**Figure 2:**
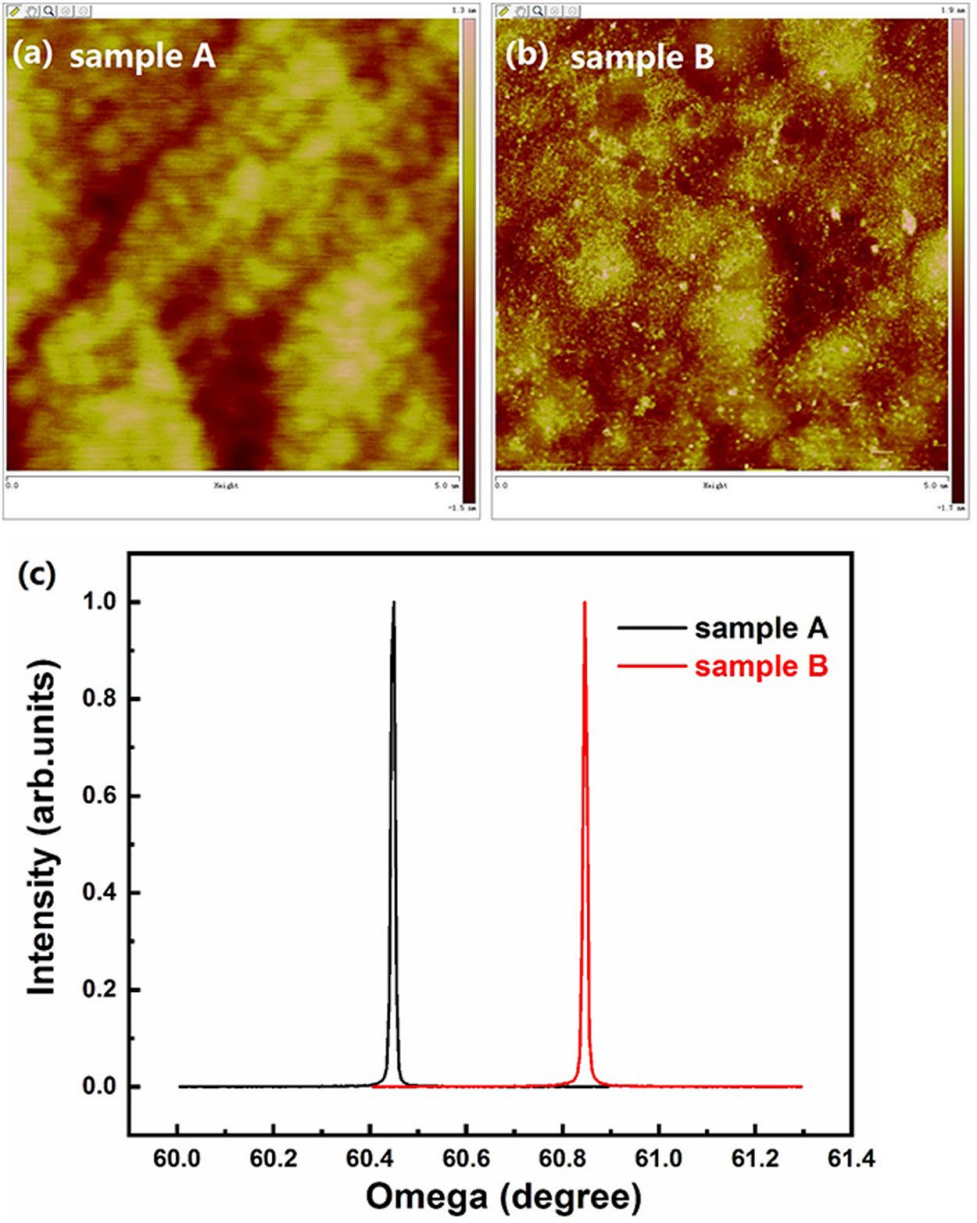
(**a**,**b**) show AFM results of sample A and B, respectively; (**c**) show XRD results of sample A and B.

The quality of diamond with 200 nm single crystal H-diamond was characterized by Micro-Raman and XRD. 20 × objective lens was used in Raman measurement with 532 nm excitation laser and 0.4 cm^−1^/pixel resolution. Three Raman random points were measured on sample A and B, respectively. The average Raman FWHM results of sample A and B were 4.2 cm^−1^ and 4.0 cm^−1^, respectively. Rocking curves of sample A and B were measured by using four-bounce Ge (2 2 0)-monochromated Cu-K𝛼, with a 10 mm slit on the detector arm. The XRD FWHM of sample A and B were 0.012° and 0.011°, shown as Fig. 2(c). The roughness of sample A and B were 0.32 and 0.37 nm measured by AFM, shown as Fig. 2(a,b), respectively.

**Figure 3:**
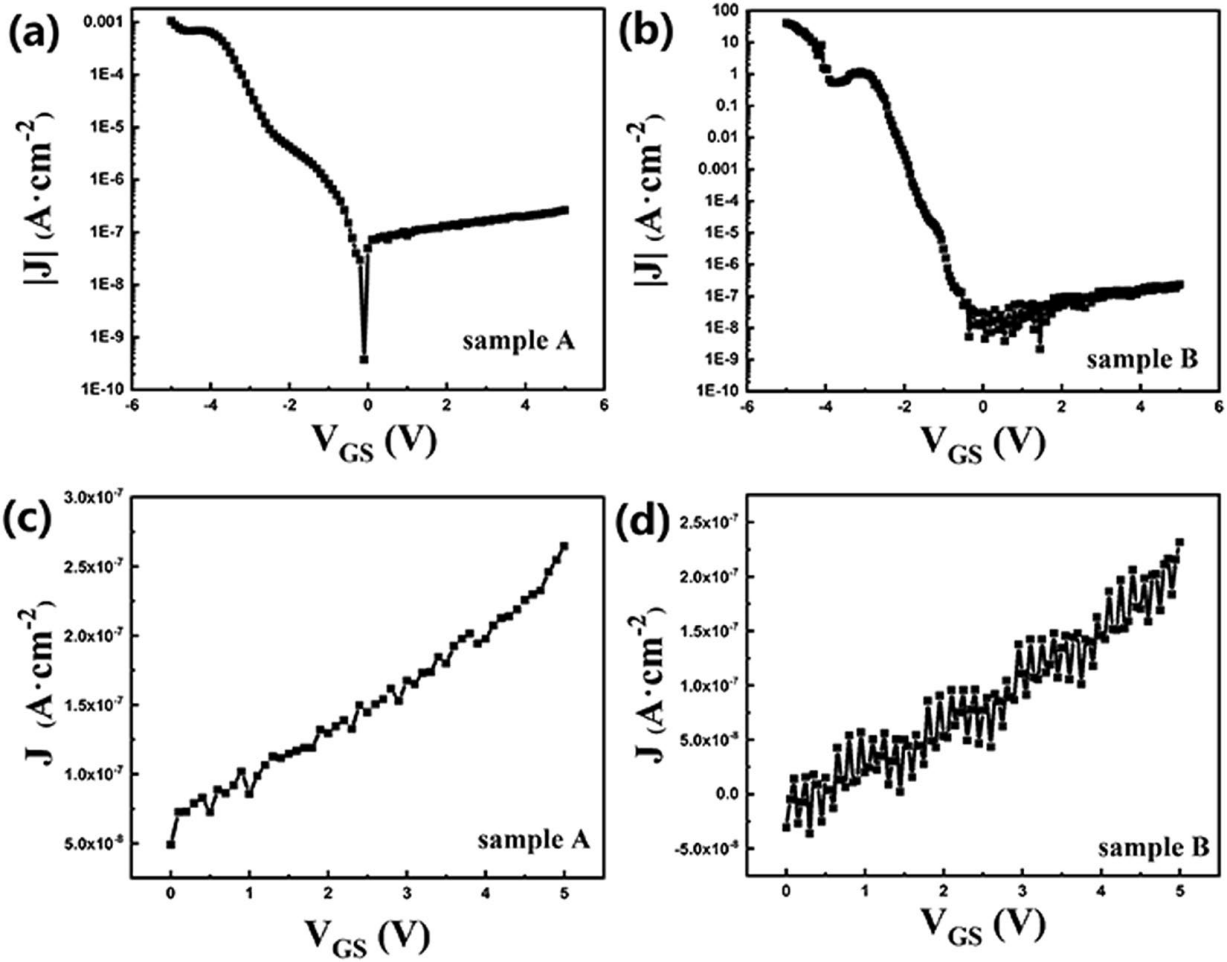
(**a**,**b**) show the absolute value of gate leakage current density (|J|) of sample A and B in the log coordinate from −5 to 5 V, respectively; (**c**,**d**) show the J of sample A and B from 0 to −5 V, respectively.

Figure 3(a,b) show the absolute value of gate leakage current density (|J|) of sample A and B in the log coordinate, respectively. Figure 3(c,d) show the gate leakage current density (J) of sample A and B, respectively. In Fig. 3(a,b), the gate-source voltages (V_GS_) was from −5 to 5 V. In Fig. 3(c,d), the V_GS_ was from 0 to 5 V. When V_GS_ decreased from 5 to 0 V, as shown in Fig. 3(c,d), the J decreased from 2.6 × 10^−7^ to 5 × 10^−8^ A·cm^−2^ for sample A and from 2.3 × 10^−7^ to −3 × 10^−8^ A·cm^−2^ for sample B, indicating that J of sample A and B were almost the same. When V_GS_ decreased from 0 to −5 V in Fig. 3(a,b), J decreased from 5 × 10^−8^ to −1.0 × 10^−3^ A·cm^−2^ for sample A and from −3 × 10^−8^ to −40 A·cm^−2^ for sample B. And also, when V_GS_ was −5 V, the J ratio between sample B and A was 4 × 10^4^. It shows that the J of sample A was much smaller than that of sample B at V_GS_ ranging between 0 and −5 V, indicating that AlO_x_ dielectric layer in sample A could effectively reduce leakage current.

**Figure 4:**
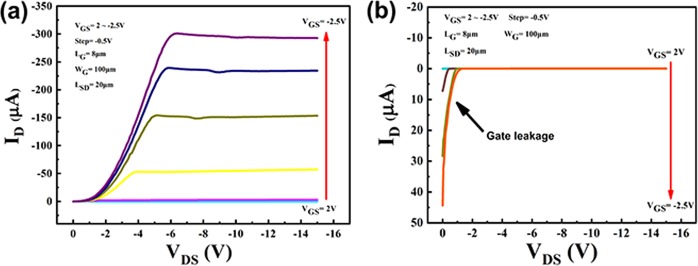
Results of output characteristic curves of (**a**) sample A and (**b**) sample B.

Figure 4 shows drain-source current (I_D_) versus drain-source voltage (V_DS_) output characteristics (I_D_ − V_DS_) curves of (a) sample A and (b) sample B. In Fig. 4(a,b), the V_GS_ was changed from 2 to −2.5 V in steps of −0.5 V. When V_GS_ = 2, 1.5, 1, 0.5, 0 and −0.5 V, the curves of I_D_ could not be distinguished due to the small value of them in Fig. 4(a). Base on the measurement results of sample A in Fig. 4(a), the I_D_ shows the distinct pinch-off characteristic. And also, p-type channel characteristics were observed in Fig. 4(a), because the value of I_D_ decreased with the decreasing of V_DS_ value from 2 to −2.5 V^3^. When the V_GS_ = −2.5 V and V_DS_ = −6.4 V, I_D_ was 300.9 μA, which was the maximum I_D_ value in Fig. 4(a). When the V_GS_ = 0 V and V_DS_ = −6.4 V, I_D_ was −5.6 × 10^−6^ μA (data not shown), indicating that on/off ratio was about 5.4 × 10^7^. In Fig. 4(b), when V_DS_ = 0 V, the reverse (positive) I_D_ was observed, as indicated by the black arrow. And then, the value of I_D_ decreased with the valve of V_DS_ decreasing. The reason for the reverse I_D_ was the gate leakage current according to *Ricardo S*. *Sussmann*^19^. This leakage current could be caused by defects on diamond surface^19^. In Fig. 4(a), no reverse I_D_ was observed, indicating that gate leakage current was small in sample A, which was agreement with the results in Fig. 3. Therefore, AlO_x_ dielectric layer in sample A can prevent reverse I_D_ in I_D_ − V_DS_ measurement.

**Figure 5:**
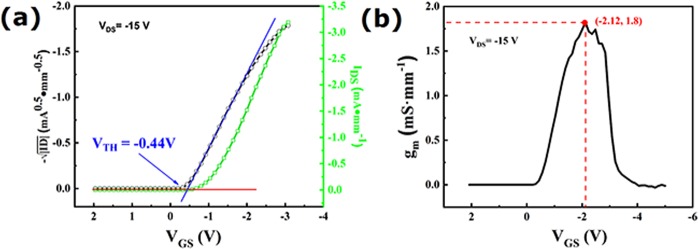
(**a**) Result of transfer characteristic curves of sample A; (**b**) transconductance of sample A.

The transfer characteristics of sample A was measured to evaluate the threshold voltage (V_TH_) and maximum of extrinsic transconductance (g_m_), as shown in Fig. 5(a,b), respectively. In Fig. 5(a), the V_TH_ was −0.44 V at the V_DS_ = −15 V calculated by method of *Jiangwei Liu*^15^, indicating that FET with AlO_x_ dielectric layer showed enhancement mode (normally-off), which has been discussed in our previous report^6^. One reason for normally-off characteristics is decreasing of hydrogen-termination on diamond surface during fabrication process of sample A, such as photolithographic and EB process. Another reason could be the depletion of hole carriers in FET under channel, which is due to the large difference of work function between 100 nm Al gate and H-diamond^6^. In Fig. 5(b), the g_m_ was 1.8 mS·mm^−1^ at V_DS_ = −15 V and V_GS_ = −2.1 V.

In our previous work, normally-off H-diamond FET had been carried out with 3 nm Al_2_O_3_ dielectric layer^6^. However, in this work, H-diamond FET with 6 nm AlO_x_ has been realized. For the previous one, 3 nm discontinuous Al_2_O_3_ film was formed by thermally oxidizing 3 nm Al in the air. During the long time thermally oxidization and gate electrode deposition process, parts of adsorbate and hydrogen-termination between Al_2_O_3_ gaps on H-diamond could be reduced^18,20^. While for this work, 6 nm continuous AlO_x_ film was formed by auto-oxidizing 6 nm Al film at RT, and could protect adsorbate and hydrogen-termination during gate electrode deposition process^18,20^.

## Conclusions

In summary, fabrication of H-diamond FETs with AlO_x_ dielectric layer formed by auto-oxidizing 6 nm Al layer in the air at RT has been successfully carried out. The leakage current density in FET with AlO_x_ dielectric layer was 4 × 10^4^ lower than that without AlO_x_ dielectric layer at V_GS_ = −5 V. The AlO_x_ dielectric layer can reduce leakage current and prevent reverse I_D_ in I_D_ − V_DS_ caused by defects on diamond surface. The on/off ratio of FET with AlO_x_ dielectric layer was 5.4 × 10^7^. FET with AlO_x_ dielectric layer has shown normally-off characteristics with −0.44 V threshold voltage at V_DS_ = −15 V.
